# A genuine need or nice to have? Understanding HTA representatives’ perspectives on the use of patient preference data

**DOI:** 10.1017/S026646232400463X

**Published:** 2024-11-20

**Authors:** Evi Germeni, Simon Fifer, Mickaël Hiligsmann, Barry Stein, Mandy Tonkinson, Maya Joshi, Alissa Hanna, Barry Liden, Deborah A. Marshall

**Affiliations:** 1 Health Economics and Health Technology Assessment (HEHTA), School of Health and Wellbeing, University of Glasgow, Glasgow, UK; 2 Community and Patient Preference Research (CaPPRe), Sydney, New South Wales, Australia; 3 Department of Health Services Research, CAPHRI Care and Public Health Research Institute, Maastricht University, Maastricht, The Netherlands; 4 Colorectal Cancer Canada (CCC), Montreal, Quebec, Canada; 5 Public Involvement Programme, National Institute for Health and Care Excellence (NICE), Manchester, UK; 6 Patient Engagement, Edwards Lifesciences, Irvine, CA, USA; 7 Leonard D. Shaeffer Center for Health Policy and Economics, University of Southern California, Los Angeles, California, USA; 8 Department of Community Health Sciences, Cumming School of Medicine, University of Calgary, Calgary, Alberta, Canada

**Keywords:** health technology assessment, patient preferences, patient participation, stakeholder engagement

## Abstract

**Objectives:**

The roles and potential value of patient preference (PP) data in health technology assessment (HTA) remain to be fully realized despite an expanding literature and various efforts to establish their utility. This article reports lessons learned through a series of collaborative workshops with HTA representatives, organized by the Health Technology Assessment International’s Patient Preferences Project Subcommittee.

**Methods:**

Five online workshops were conducted between June 2022 and June 2023, seeking to facilitate collaborative learning and reflection on ways that PP data can be integrated into HTA. Participants included nine HTA representatives from the United States, Canada, Australia, England, and the Netherlands. Workshops were recorded, transcribed, and thematically analyzed.

**Results:**

Despite appreciating the value of PP data, participants were ambivalent about their use in HTA. Some felt that they were already getting the information they needed from the cost-effectiveness analysis or existing patient involvement processes. Others thought that PP data would be very helpful at the initial and final stage of the decision-making process and, particularly, in the following cases: (a) when technology has important non-health benefits; (b) when the clinical and/or cost-effectiveness evidence is marginal; and (c) when treatment is indicated for a large and heterogeneous population. Issues related to the validity and reliability of PP studies were frequently raised, with preference heterogeneity at the core of these concerns.

**Conclusions:**

Collaborating with HTA representatives in the “co-creation” of PP research can help address their concerns and facilitate mutual learning about how PP data can be used in HTA.

## Introduction

Despite a growing impetus to incorporate the “patient voice” into health technology assessment (HTA), the degree to which this has been achieved remains unclear (1–3). In many countries, patients have now secured a seat at the decision-making table, with their knowledge and expertise no longer contested (4). Patients (and patient organizations) provide unique, experiential information about what it is like to live with a disease and offer a much-needed, real-life view of the potential benefits and risks of new technologies. Yet, even in countries that have implemented considerable patient involvement efforts, concerns still exist as to whether these are adequate or fully capture the diversity and breadth of patient experience. Increasing and broadening patient input through more scientific means is gradually being seen as a way to make HTA more responsive to patient needs (5, 6).

Health preference research has been gaining traction as a potential avenue for systematically incorporating patients’ perspectives, needs, and experiences into HTA decision-making (7–9). The U.S. Food and Drug Administration defines “patient preference information” as “qualitative or quantitative assessments of the relative desirability or acceptability to patients of specified alternatives or choices among outcomes or other attributes that differ among alternative health interventions” (10). In this context, qualitative assessments involve the use of preference exploration methods, such as semi-structured interviews and focus groups (11), whereas quantitative assessments typically rely on the use of preference elicitation techniques through trade-offs, such as discrete choice experiments (DCEs) and best-worst scaling (BWS) (12). Quantitative assessments, which are the focus of this article, quantify preferences using statistical analysis and allow for the detection of preference heterogeneity among patients (13).

Key stakeholders, including regulatory authorities and HTA bodies, are increasingly recognizing the value of patient preference (PP) data, yet these are still far from routinely incorporated in assessments and decision-making (14, 15). Based on the results of a systematic literature review, a number of issues are currently hindering the successful integration of PP data into HTA, which could fall into five main categories: (a) conceptual – pertaining to the definition and characterization of PP; (b) normative – concerning whose preferences should be elicited (e.g., patients with or without treatment experience, patient representatives, and carers); (c) procedural – focusing on ways to integrate PP data into the existing procedures of HTA; (d) methodological – focusing on establishing good research practice in the field; and (e) practical – relating to time and resource constraints and other aspects of conducting a PP study (16).

The Patient Preferences Project Subcommittee (PPPS) of the Health Technology Assessment International’s Patient and Citizen Involvement Interest Group (HTAi PCIG) initiated a program of work focusing on better understanding existing barriers to the use of PP data in HTA and identifying solutions that could lead to increased uptake. Following an exploratory survey on the use, impact, and role of PP data in HTA (17), this article reports lessons learned through a series of collaborative workshops with HTA representatives, conducted over the course of a year.

## Methods

Five online workshops, lasting approximately 1 hr each, were conducted between June 2022 and June 2023. Workshops were presented and moderated by three members of the HTAi PCIG PPPS and sought to facilitate collaborative learning and reflection on ways that PP data can be better integrated into HTA decision-making. Key questions guiding the design of the workshops were organized under three pillars: “learn”; “grow”; and “improve” (Table 1). These were developed, reviewed, and approved by all HTAi PCIG PPPS members, including patients, drug, and device industry representatives, health technology assessors, and (academic and private) researchers with expertise in PP research.

**Table 1.** Questions guiding the design of the workshops

LEARN	What do HTA organizations need to learn to better understand patient preference studies? How can patient preferences be incorporated into HTA review? What is the value/limitations of patient preferences in the HTA review process?
GROW	How can the HTA community expand its capabilities and expertise in this field? What kind of resources need to be developed to further support HTA use of patient preferences?
IMPROVE	How can patient preference studies be better designed to reflect the needs of HTA organizations? How can patient organizations and/or industry improve the way patient preferences are gathered and submitted into the HTA process? Are there opportunities for HTA organizations to adapt their processes or reviews to better incorporate patient preferences?

### Participant Recruitment

A one-page summary providing an overview of the project, along with key objectives and time commitment required for participation, was shared with five HTA organizations, perceived by the group as most likely to have some experience or understanding of PP data. We requested that potential participants be identified on the basis of two criteria: (a) their familiarity (ideally, first-hand experience) with the HTA review process; and (b) their ability to liaise with internal HTA leadership so that they could share lessons learned from the process. A total of nine HTA representatives (one male and eight female) agreed to participate. Participants fulfilled a number of roles directly relevant to the HTA process (e.g., technical advisors, patient engagement leads, and chief scientists) and were employed at the following organizations: the English National Institute for Health and Care Excellence (NICE) (*n* = 2); the Canadian Agency for Drugs and Technologies in Health (CADTH) (*n* = 2); the Dutch National Health Care Institute (ZIN) (*n* = 2); the US Institute for Clinical and Economic Review (ICER) (*n* = 2); and the Medical Services Advisory Committee (MSAC) of the Australian Department of Health and Aged Care (*n* = 1).

### Workshop Description

The first workshop served as a kick-off to introduce all parties and explain the goals of the project. Participants were advised that all discussions would be held under the Chatham House Rule (i.e., participants would be free to use information disclosed, but neither the identity nor the affiliation of the speakers would be revealed), to allow for an open and frank exchange of views. During subsequent workshops, participants were presented with two different PP case studies. Case study 1 consisted of two treatment-agnostic DCEs and one BWS exercise, aiming to measure preferences for different colorectal cancer treatments among: (a) cancer patients (metastatic and nonmetastatic); (b) caregivers; and (c) adults without colorectal cancer. For the purpose of the workshops (and given that the study was still ongoing at the time of the workshops), the presenters focused on the simplest DCE that asked metastatic cancer patients to choose between treatment A or B on the basis of two attributes: overall survival and change in health-related quality of life during treatment. Case study 2 was a labelled DCE that asked patients with heart valve disease (HVD) to choose between an invasive HVD procedure or a minimally invasive HVD procedure, based on seven treatment attributes: risk of mortality, risk of stroke, requirement for dialysis, risk of needing a permanent pacemaker, durability of valve, independence after procedure, and out-of-pocket costs. Full details of this study are reported elsewhere (18). Workshop participants were provided with information on three core outputs from these studies, namely relative attribute importance, predicted uptake, and maximum acceptable risk. They were then posed the following questions: (1) ‘What does this evidence tell you as a decision maker?’; (2) ‘How does it align with other evidence you already consider?’; and (3) ‘Would this evidence help you in decision making?’. Participants were also asked to review this evidence as if they were committee members, and to assume that both studies had been completed, were valid and robust, and were ready to be used as evidence by the HTA committee when making recommendations. The last workshop focused on addressing HTA representatives’ previous comments and questions about preference heterogeneity, with a further case study – a DCE on myeloma treatment preferences (19) – being introduced to guide the discussion. The workshop concluded with a review of key lessons learned and scoping next steps.

### Data Analysis

With participant permission, all workshops were recorded and automatically transcribed through the Zoom video-conferencing platform. Drawing on Braun and Clarke’s approach to thematic analysis (20), data analysis began with reading through all transcripts several times and producing data summaries for each workshop, in order to gain a good overview of all material. Participants’ perspectives on the use of PP data were subsequently coded, with similar codes grouped together to form broader categories and then initial themes. Ongoing refinements were made based on further examination of the transcripts and data summaries. The final themes were organized in such a way as to create a narrative, which was both consistent with the data and informative in relation to the study objectives.

## Results

**Figure 1.**
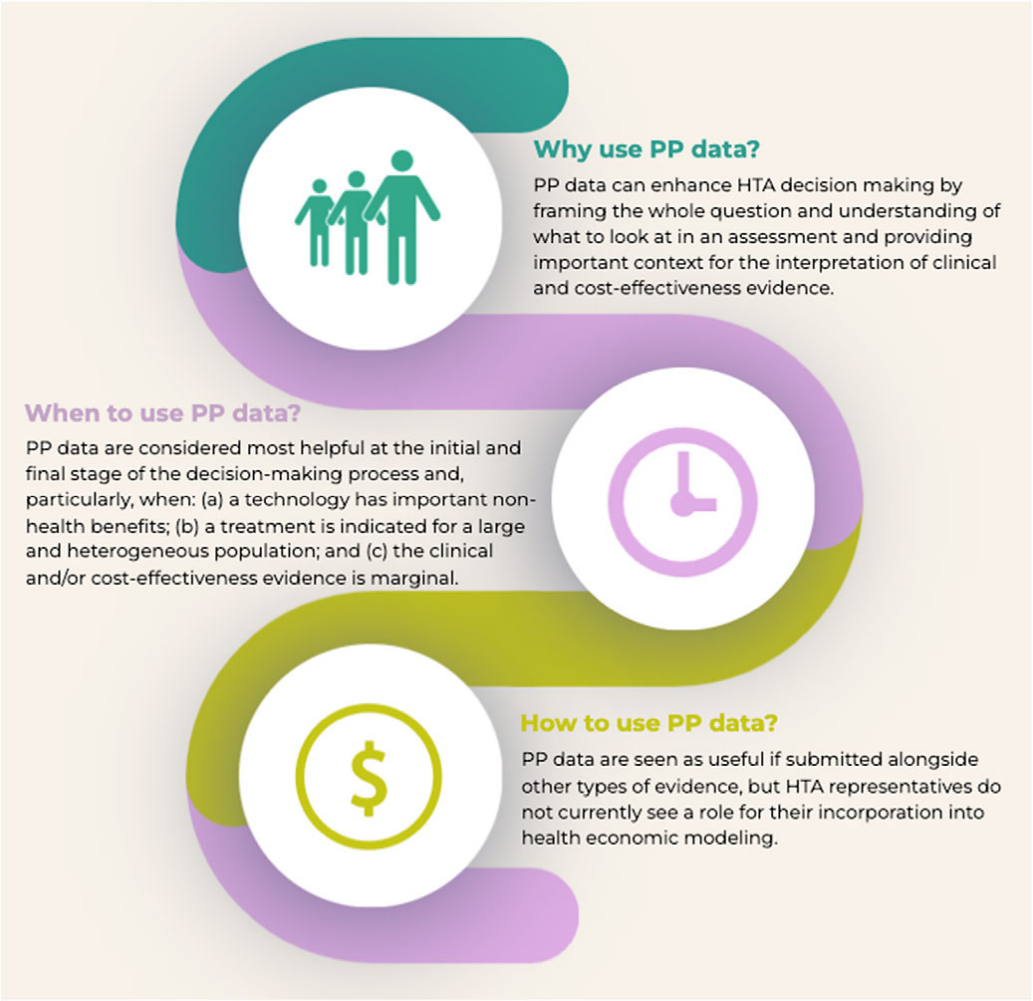
Key study findings on why, when, and how to use PP data in HTA.

We identified two main themes from the data analysis: (a) a genuine enhancement or “gilding the lily” (i.e., an unnecessary or counterproductive embellishment), capturing perceived added value of incorporating PP data into the existing procedures of HTA; and (b) the preference measurement riddle, focusing on methodological concerns that participants expressed in relation to PP studies. Identified themes, with accompanying sub-themes, are presented in Table 2 and described in detail below. Representative participant quotes are provided throughout for illustrative purposes and to promote the transparency and rigor of data analysis. To protect participants’ anonymity, all identifying information has been removed from the quotes; participant numbers are used to differentiate voices. Participant responses on why, when, and how to use PP data in HTA are summarized in Figure 1.

**Table 2.** Main themes and sub-themes identified from the data analysis, along with representative participant quotes

Themes	Subthemes	Representative quotes
A genuine enhancement or “gilding the lily”?	Usefulness of PP data	*“I think the usefulness of this sort of information is perhaps situational and dependent on the type of study that has been undertaken. So, I do think that, in theory, a study like this could be useful in some situations, possibly less so in others, but overall useful to the committee in sort of enhancing their decision rather than being the fundamental basis for their decision making.”*
	HTA stage	***“**This is all very useful. Particularly I think in the scoping phase of an assessment, for example, where we need to think about which outcomes we want to see, and what kind of differences we would like to see in those outcomes, in order to talk about a relevant improvement.”*
	Areas of added value	*“I usually look at highly specialized technologies, where they’re like one or two patients in the entire country, so it’s really not worth doing that sort of thing for that. But if it’s huge populations with really prevalent diseases, I don’t know, osteoporosis or something, then I could see the value of taking this approach to really sort of quantify what the really important patient preferences are. But, otherwise, it just seems like too much.”*
	Incorporation within QALY	*“So, you know, even if we get to a point where we can work more strongly with patient communities and life sciences manufacturers to generate these data, how do we incorporate that into our methods?”*
The preference measurement riddle	Whose preferences	*“In a project that we’re doing, we’re looking at a pediatric rare disease, and it’s the case that parents actually value longer survival more than children might do. So, you know, not to make it even more complicated, but I just wanted to put that out there. Because I had never thought of that before, like who is valuing this longer survival versus quality of life? There might be different perspectives.”*
	Fluidity of preferences	*“How can you be that precise with something that is so fluid and relates to patients?”*
	Preference heterogeneity	*“If it’s super clear that this is hugely important to the patients and all patients agree, and it’s really consistent, then that would have more weight instead of ‘well, half of patients think this’. If it’s really all over the place, that is not as impactful, I would argue… But if we could have a consensus that quality of life is more important than survival from a really large survey, and have the backup of the patient organizations, that allows decisions to start being made more easily.”*

### A Genuine Enhancement or “Gilding The Lily”?

The majority of the workshop participants reported that they did not have direct experience with PP data: *“We just don’t get this data. Nobody is submitting it. So, you know, it’s not really a philosophical matter of whether we would use it or not. It’s like we kinda [kind of] need somebody to bring it to us, to even make a determination if it’s something we could use”* (participant 3). Despite appreciating the value of such data (sometimes, though, in a broader healthcare context), they seemed ambivalent about their use in HTA decision-making. Some stated that they were already getting the information they needed from the cost-effectiveness analysis or from existing patient involvement processes; therefore, investing time and money into PP studies might be unnecessary: *“I think this would be really helpful in certain situations, not across the board in terms of what we do, just because I do think that we get what we need out of our patient involvement at the moment. And we also filter out conflicts pretty well. I guess the question is how much more benefit will we get from doing something that will potentially cost quite a lot, and take a lot of time, and could potentially impact the timelines for the appraisal process if we wanted to give, you know, whoever it is that’s putting together this evidence, the time to actually collect it. Because, obviously, we have pressure for trying to be as timely as possible with our guidance. So, I guess it’s almost like, are we ‘gilding the lily’ by doing so much more?”* (participant 1).

Others thought that evidence from PP studies is more systematic than direct patient input and therefore would be particularly helpful either at an initial or at the final stage of the decision-making process: *“I see the biggest value of this being in that upfront consideration, where we’re really trying to ground ourselves in an understanding of what’s most important to patients in terms of their outcomes and pursuit of life goals and all that kind of information… The other place that this comes in for us, and where I think this is hugely helpful, is in our public deliberations, where there is more of a discussion around benefits beyond health and trying to get at those things that are not well captured in the economic models and are really important to patients”* (participant 3). Apart from cases where technologies might have important non-health benefits, other examples where PP studies could add value to the assessments were discussed. These included cases where treatment is indicated for a large population (e.g., a treatment for osteoporosis, as opposed to a treatment for a very rare disease, where the opportunities for quantitative methods to explore preference heterogeneity are naturally increased), as well as cases where the clinical and/or cost-effectiveness evidence is marginal: *“I think it just helps to give context… And it helps to sway us if we’re not sure whether or not the effectiveness is enough, or whether it’s cost effective enough”* (participant 8).

Although participants typically struggled to see an immediate role for the incorporation of PP data into health economic modeling, most of them agreed that PP evidence would enhance decision-making if submitted alongside other types of evidence *“Sometimes feeding this information in, you have to understand that it frames the whole question and understanding of what we’re looking at in the assessment. Even if, you know, the data actually being analyzed are still coming from clinical trials, because that’s the drug under review. So, I think it’s important to communicate that better, in a way that’s understandable, and to demonstrate that there was impact from those studies, even if it doesn’t necessarily go into an economic model per se”* (participant 3). The view that the comprehensiveness of this sort of information would get diluted in an incremental cost-effectiveness ratio was also expressed: *“So, where would this information get embedded? I guess it would go into the health economic analysis, right? For the QALY discussion. But I’m wondering if it needs to go somewhere else… I’m just thinking about the breadth or the depth of this kind of work that is much more sophisticated than, you know, asking people what their utility value is on the EQ-5D, right?”* (participant 5).

### The Preference Measurement Riddle

Apart from discussions of ways in which PP data could be better incorporated into the existing procedures of HTA, participants very often brought up methodological queries and concerns, such as whose preferences should be measured (e.g., the preferences of patients or those of the general public), or how stable preferences are over time: *“I think this is really relevant stuff for HTA agencies, but I was just wondering whether you have any information on how stable those preferences are… Because I think it’s not ethically feasible to ask patients at the end of their life, ‘Would you make the same decision that you did make? Was it worth it?’ I mean, do these preferences change over time when people have experience with side effects in reality?”* (participant 6). The fluidity and heterogeneity of patient preferences were often seen as prohibiting factors for the use of such data in an HTA context: *“I am just thinking that preferences might change throughout your life… And preferences might be different for different groups of patients… So, I am not sure. I just think it’s difficult [to use PP data in HTA]. I mean, you wouldn’t want as an agency to be saying, ‘Well, we recommend treatment B, because most people prefer it’. But I think it might be very useful to patients if they’re trying to make decisions between one treatment and another at the same place in the treatment pathway”* (participant 2).

Accounting for preference heterogeneity in the analysis of PP data was also met with relative skepticism, with participants commenting on the complexity of these techniques and the need for better payer education: *“The education of the payers is always important. So, I love the data that you presented, but one thing that’s in my mind is, how do we continue to have these discussions with the payers and educate them about different techniques? Because these are sometimes quite complex… And, you know, you’ve done like a great job with the data visualization and all, but I can already see with the word ‘segmentation’, for instance, everybody going, ‘Oh, I don’t know how to deal with this”* (participant 5). Fears that subgroup analysis might lead to widening inequalities were also voiced: *“One thing that we’re very wary of, with our new health equity initiative, is, you know, our end goal for the report is essentially to recommend a fair price at a population level for the treatment under review. And one thing we don’t want to do is say that, you know, for this class of people, that treatment is going to end up being more expensive or less expensive if we kind of do that sub-population analysis. We definitely want to stay away from that, from that part of the analysis… So, I’m kind of curious how preferences and all that [segmentation data] fits together and not sort of [lead to] making recommendations that might in some way, unintended way, devalue one group over the other, as we’re recommending prices for treatments”* (participant 9).

## Discussion

Understanding HTA representatives’ perspectives and concerns about the use of PP data is essential for the future of these discussions. In a similar focus group study with HTA representatives from Germany, Belgium, and Canada, van Overbeeke et al. (13) remarked that the majority of their participants did not have expertise in PP studies, concluding that investment in the training of HTA representatives might be necessary. Indeed, our decision to organize and conduct collaborative workshops with HTA representatives was motivated by an apparent need to increase awareness about PP data and their potential contribution to HTA decision making. Although our participants came from HTA organizations that have implemented considerable patient involvement efforts, most of them also reported that they did not have direct experience with PP data, which might explain some of the concerns raised here.

Similar to Huls et al. systematic review of challenges to using PP data in HTA (16), issues of procedural and methodological nature were most frequently raised during our workshops. Common procedural issues concerned whether PP data should be incorporated within the quality-adjusted life-years (QALY), at what stage of the decision-making process PP evidence should be considered, and in which contexts such data would be most helpful. In line with existing evidence and a recent commentary published by NICE (13, 21), our participants struggled to see an immediate role for the incorporation of PP data within the QALY, but most of them agreed that PP evidence would enhance decision making if submitted alongside other types of evidence. Based on their perspectives, PP data would be most helpful at the initial, scoping phase of an assessment and during the deliberation process, and would particularly add value in the following cases: (a) when the clinical and/or cost-effectiveness evidence is marginal; (b) when technology has important non-health benefits; and (c) when a treatment is indicated for a large and heterogeneous population.

Methodological issues most frequently raised by HTA representatives participating in this study mainly focused on the reliability and validity of PP studies, with the fluidity and heterogeneity of patient preferences at the core of these concerns. Although this is consistent with existing findings (16, 22), it is interesting that it still emerged, given that participants were instructed to share their views on the basis that presented case studies “were valid and robust, and ready to be used as evidence by the HTA committee”. Improving the validity and reliability of PP studies has been found to be a key priority among the academic community (23) and, despite the noticeable growth of relevant publications over the last few years (24–26), HTA representatives’ concerns might reflect broader academic debates. Certain issues, however, raised in the context of our workshops have not been previously reported and might warrant further attention. In particular, accounting for preference heterogeneity in the analysis of PP data was met with skepticism by some of our participants, who commented on the complexity of these techniques and expressed fears that subgroup analysis might unintentionally lead to widening inequalities. In the Whichello et al. interview study (22), preference heterogeneity was viewed by participating stakeholders as a “gift”, because it enables subgroup identification and exploration of opinions. Yet, their participants also posited that estimating heterogeneity is not straightforward and often difficult to integrate into decision making. Research into the relative costs and benefits of understanding PP heterogeneity could be helpful in addressing this conflict. Notwithstanding the challenges of incorporating preference heterogeneity into HTA decision making, turning a blind eye to such information is also inappropriate, given its potential relevance for improving health. Where the heterogeneity of patient preferences for a particular technology has been demonstrated, reimbursement decision making should incorporate the implications of that heterogeneity in its determinations, with separate recommendations for patient groups with different preferences. Employing average assessments in such instances will not lead to optimized outcomes for patients.

Practical challenges related to the use of PP data in HTA were also discussed among our participants, including the time and resources required to conduct PP studies and the potential impact that this might have on the timelines for the appraisal process. Indeed, based on our previously conducted survey with members of HTA bodies and affiliated organizations (17), time and resource constraints were reported as the leading reasons why PP data were not submitted in assessments. Nevertheless, results from these in-depth workshops suggest that such concerns (albeit frequently voiced) might be secondary, as they were typically expressed by individuals who in general did not seem very convinced about the added value that PP data can bring to HTA. Although the importance of these practical considerations should not be underestimated, our results indicate that priority should be given to establishing more consensus and clarity on procedural and methodological issues concerning the integration of PP data in HTA decision-making. Pragmatic guidance on how to conduct rigorous PP studies and how to include these findings in existing HTA processes will most likely extend their applicability.

To the best of our knowledge, this is the first study that directly engaged with an international and diverse sample of HTA representatives over a prolonged period of time, in order to facilitate mutual learning about how PP data can be used in HTA. Although lessons learned were crucial, it is important to remember that the nature of this work and the small study sample might restrict the generalizability of these findings. Moreover, the extent to which views expressed here represent the broader HTA stakeholder community or might be applicable beyond North America and Western Europe, is largely unknown. Our next steps include the organization of mock deliberation workshops to further advance our understanding of how HTA committees could employ PP data in real-life decision-making.

## References

[1] Perfetto EM, Oehrlein EM, Boutin M, Reid S, Gascho E. Value to whom? The patient voice in the value discussion. Value Health. 2017;20(2):286–291. 28237211 10.1016/j.jval.2016.11.014

[2] Wortley S, Wale J, Grainger D, Murphy P. Moving beyond the rhetoric of patient input in health technology assessment deliberations. Aust Health Rev. 2017;41(2):170–172. 27224935 10.1071/AH15216

[3] Gunn CJ, Regeer BJ, Zuiderent-Jerak T. A HTA of what? Reframing through including patient perspectives in health technology assessment processes. Int J Technol Assess Health Care. 2023;39(1):e27. 37198925 10.1017/S0266462323000132 PMC11570000

[4] Single ANV, Facey KM, Livingstone H, Silva AS. Stories of patient involvement impact in health technology assessments: A discussion paper. Int J Technol Assess Health Care. 2019;35(4):266–272. 31337453 10.1017/S0266462319000552

[5] Facey KM, Bedlington N, Berglas S, Bertelsen N, Single ANV, Thomas V. Putting patients at the centre of healthcare: Progress and challenges for health technology assessments. Patient. 2018;11(6):581–589. 30051315 10.1007/s40271-018-0325-5

[6] Tegenge MA, Moncur MM, Sokolic R, Forshee RA, Irony T. Advancing the science of patient input throughout the regulatory decision-making process. Learn Health Syst. 2017;1(3):e10032. 31245564 10.1002/lrh2.10032 PMC6508573

[7] Marsh K, van Til JA, Molsen-David E, Juhnke C, Hawken N, Oehrlein EM, et al. Health preference research in Europe: A review of Its use in marketing authorization, reimbursement, and pricing decisions-report of the ISPOR stated Preference Research Special Interest Group. Value Health. 2020;23(7):831–841. 32762984 10.1016/j.jval.2019.11.009

[8] Berglas S, Jutai L, MacKean G, Weeks L. Patients’ perspectives can be integrated in health technology assessments: An exploratory analysis of CADTH Common Drug Review. Res Involv Engagem. 2016;2:21. 29062521 10.1186/s40900-016-0036-9 PMC5611639

[9] Janssens R, Barbier L, Muller M, et al. How can patient preferences be used and communicated in the regulatory evaluation of medicinal products? Findings and recommendations from IMI PREFER and call to action. Front Pharmacol. 2023;14:1192770. 37663265 10.3389/fphar.2023.1192770 PMC10468983

[10] Patient Preference Information - Voluntary Submission, Review in Premarket Approval Applications, Humanitarian Device Exemption Applications, and De Novo Requests, and Inclusion in Decision Summaries and Device Labeling Guidance for Industry, Food and Drug Administration Staff, and Other Stakeholders. 2016; Available from: https://www.fda.gov/regulatory-information/search-fda-guidance-documents/patient-preference-information-voluntary-submission-review-premarket-approval-applications.

[11] Germeni E, Szabo S. Beyond clinical and cost-effectiveness: The contribution of qualitative research to health technology assessment. Int J Technol Assess Health Care. 2023;39(1):e23. 37092753 10.1017/S0266462323000211 PMC11570152

[12] Soekhai V, Whichello C, Levitan B, et al. Methods for exploring and eliciting patient preferences in the medical product lifecycle: A literature review. Drug Discov Today. 2019;24(7):1324–1331. 31077814 10.1016/j.drudis.2019.05.001

[13] van Overbeeke E, Forrester V, Simoens S, Huys I. Use of patient preferences in health technology assessment: Perspectives of Canadian, Belgian and German HTA Representatives. Patient. 2021;14(1):119–128. 32856278 10.1007/s40271-020-00449-0 PMC7794204

[14] Benz HL, Saha A, Tarver ME. Integrating the voice of the patient into the medical device regulatory process using patient preference information. Value Health. 2020;23(3):294–297. 32197723 10.1016/j.jval.2019.12.005

[15] Mott DJ. Incorporating quantitative patient preference data into healthcare decision making processes: Is HTA falling behind? Patient. 2018;11(3):249–252. 29500706 10.1007/s40271-018-0305-9

[16] Huls SPI, Whichello CL, van Exel J, Uyl-de Groot CA, de Bekker-Grob EW. What Is next for patient preferences in health technology assessment? A systematic review of the challenges. Value Health. 2019;22(11):1318–1328. 31708070 10.1016/j.jval.2019.04.1930

[17] Hiligsmann M, Liden B, Beaudart C, et al. HTA community perspectives on the use of patient preference information: Lessons learned from a survey with members of HTA bodies. Int J Technol Assess Health Care. 2024;40(1):e17. 38439624 10.1017/S0266462324000138 PMC11569952

[18] Fifer S, Keen B, Guilbert-Wright P, Yamabe K, Murdoch DJ. Patient preferences for heart valve disease intervention. Health Expect. 2024;27(1):e13929. 38050462 10.1111/hex.13929 PMC10726282

[19] Fifer S, Galinsky J, Richard S. Myeloma Patient Value Mapping: A Discrete Choice Experiment on Myeloma Treatment Preferences in the UK. Patient Prefer Adherence. 2020;14:1283–1293. 32801659 10.2147/PPA.S259612 PMC7395685

[20] Braun V, Clarke V. Using thematic analysis in psychology. Qual Res Psychol. 2006;3:77–101.

[21] Bouvy JC, Cowie L, Lovett R, Morrison D, Livingstone H, Crabb N. Use of patient preference studies in HTA decision making: A NICE perspective. Patient. 2020;13(2):145–149. 31942698 10.1007/s40271-019-00408-4

[22] Whichello C, van Overbeeke E, Janssens R, et al. Factors and situations affecting the value of patient preference studies: Semi-structured interviews in Europe and the US. Front Pharmacol. 2019;10:1009. 31619989 10.3389/fphar.2019.01009 PMC6759933

[23] Janssens R, Russo S, van Overbeeke E, et al. Patient preferences in the medical product life cycle: What do stakeholders think? Semi-structured qualitative interviews in Europe and the USA. Patient. 2019;12(5):513–526. 31222436 10.1007/s40271-019-00367-w PMC6697755

[24] Janssen EM, Marshall DA, Hauber AB, Bridges JFP. Improving the quality of discrete-choice experiments in health: How can we assess validity and reliability? Expert Rev Pharmacoecon Outcomes Res. 2017;17(6):531–542. 29058478 10.1080/14737167.2017.1389648

[25] Vass C, Boeri M, Karim S, et al. Accounting for preference heterogeneity in discrete-choice experiments: An ISPOR Special Interest Group Report. Value Health. 2022;25(5):685–694. 35500943 10.1016/j.jval.2022.01.012

[26] Bridges JFP, de Bekker-Grob EW, Hauber B, et al. A roadmap for increasing the usefulness and impact of patient-preference studies in decision making in health: A good practices report of an ISPOR task force. Value Health. 2023;26(2):153–162. 36754539 10.1016/j.jval.2022.12.004

